# Syndromic monogenic diabetes genes should be tested in patients with a clinical suspicion of MODY

**DOI:** 10.2337/db21-0517

**Published:** 2022-03-01

**Authors:** Kevin Colclough, Sian Ellard, Andrew Hattersley, Kashyap Patel

**Affiliations:** 1 Exeter Genomics Laboratory, Royal Devon and Exeter NHS Foundation Trust, Exeter, U.K.; 2 Institute of Biomedical and Clinical Science, University of Exeter Medical School, Exeter, U.K.

## Abstract

At present, outside of infancy, genetic testing for monogenic diabetes is typically for mutations in MODY genes that predominantly result in isolated diabetes. Monogenic diabetes syndromes are usually only tested when this is supported by specific syndromic clinical features. It is not known how frequently patients with suspected MODY have a mutation in a monogenic syndromic diabetes gene and thus missed by present testing regimes.

We performed genetic testing of 27 monogenic diabetes genes (including 18 associated with syndromic diabetes) for 1280 patients with a clinical suspicion of MODY from routine clinical care that were not suspected of having monogenic syndromic diabetes. We confirmed monogenic diabetes in 297 (23%) patients. Mutations in 7 different syndromic diabetes genes accounted for 19% (95%CI 15-24%) of all monogenic diabetes. The mitochondrial m.3243A>G and mutations in *HNF1B* were responsible for the majority of mutations in syndromic diabetes genes. They were also the 4^th^ and 5^th^ most common causes of monogenic diabetes overall. These patients lacked typical features and their diabetes phenotypes overlapped with non-syndromic monogenic diabetes patients. Syndromic monogenic diabetes genes (particularly m.3243A>G and *HNF1B*) should be routinely tested in patients with suspected MODY that do not have typical features of a genetic syndrome.

## Introduction

Maturity Onset Diabetes of the Young (MODY) is an autosomal dominant form of monogenic diabetes diagnosed outside of infancy. Mutations in *GCK*, *HNF1A* and *HNF4A* are the most common causes of MODY. The genetic diagnosis is important for determining the most effective treatment. Patients with *HNF1A* and *HNF4A* MODY are better treated with sulphonylurea whereas *GCK* MODY does not require treatment (1; 2). MODY is suspected in non-obese individuals with young-onset diabetes which does not require insulin treatment, lack islet autoantibodies and have persistent endogenous insulin (3). Syndromic forms of monogenic diabetes are less common and characterised by young-onset diabetes but unlike MODY, they typically present with additional non-autoimmune extra-pancreatic features. These syndromes are caused by mutations that can be autosomal dominant (e.g. *HNF1B*), mitochondrial (e.g. m.3243A>G) and autosomal recessive (e.g. *WFS1*). For example, a patient with an *HNF1B* mutation will commonly have diabetes and renal structural features such as renal cysts, hypoplasia and aplasia. Patients with the mitochondrial mutation m.3243A>G commonly have diabetes and bilateral sensorineural deafness (4–6). Patients with syndromic diabetes typically have a similar diabetes phenotype to MODY (young-onset diabetes, non-obese and have negative islet autoantibodies) but unlike MODY, they are more likely to be insulin treated (7). Knowledge of the specific subtype has implications for clinical management, disease prognosis, surveillance for extra-pancreatic conditions and genetic counselling for recurrence risk.

At present, outside of infancy, genetic testing for monogenic diabetes focusses on MODY genes. Genetic testing for a syndromic diabetes gene is usually undertaken only when the patient presents with characteristic clinical features suggestive of the syndrome (e.g. m.3243A>G testing if the patient has a personal or maternal family history of diabetes, deafness and other mitochondrial disease features). This testing strategy is reflected by the lack of comprehensive inclusion of syndromic diabetes genes (with the exception of *HNF1B*) in gene panels for MODY testing in the NCBI gene testing registry (8).

Monogenic syndromic diabetes has variable expressivity of additional syndromic features and can present with isolated diabetes (9–12). This in conjunction with an overlap of the diabetes phenotype with MODY may result in patients being referred from routine clinical practice for MODY testing rather than testing for a specific monogenic syndrome (13). However, the proportion of patients with suspected MODY that have a mutation in a syndromic diabetes gene is not known. A high proportion would support changing the current genetic testing strategy to include all syndromic diabetes genes on MODY gene panels whereas a low proportion would support the current testing strategy of only testing a syndromic diabetes gene if the related clinical features are present.

In this study, we analysed syndromic diabetes genes in a large cohort of patients with suspected MODY in routine clinical care to determine whether syndromic genes should be routinely tested in patients with suspected MODY.

## Methods

### Study cohort

We studied 1280 unrelated probands who were referred by UK clinicians from routine clinical care for MODY genetic testing at the Exeter Genomics Laboratory, England from 31/11/2011 to 31/11/2018. This represents all probands referred for targeted Next Generation Sequencing (tNGS) for MODY over this time period. Clinical and biological characteristics and family history were provided by clinicians at time of referral. The suspicion of a MODY diagnosis was made by the referring clinician. In all cases the referring clinician did not suspect a diagnosis of a monogenic diabetes syndrome and the clinical features provided by the clinician at time of testing did not support genetic testing for a specific monogenic diabetes syndrome.

As a comparison cohort we included 50 patients with an *HNF1B* mutation and 54 with m.3243A>G who were referred to the Exeter Genomics Laboratory over the same time period from routine clinical care with a suspicion of having the respective monogenic diabetes syndrome by the referring clinician.

All probands gave informed consent for genetic studies and approved by the North Wales ethics committee (no. 17/WA/0327). The study was performed in accordance with the principles of the Declaration of Helsinki.

### Genetic testing

We performed genetic testing for 27 monogenic diabetes genes including the m.3243A>G mutation and 17 other syndromic diabetes genes (Supplementary Table 1). The coding regions, 50 nucleotides of flanking intronic sequence of the genes and the mtDNA nucleotide m.3243 were analysed for single nucleotide variants (SNV), indels and gene deletions by targeted Next Generation Sequencing (tNGS). Our assay did not target any other mitochondrial mutations or structural rearrangements. We used the Agilent SureSelect custom capture library and an Illumina NetSeq 500 NGS sequencing platform according to the methodology described by Ellard et al. (13). Our assay sequenced 99.7% of bases within the regions of interest at a minimum 30x read depth for all patients. All sequence variants are described using the nomenclature guidelines recommended by the Human Genome Variation Society (HGVS) (3). Interpretation and classification of sequence variants was undertaken based on the American College of Medical Genetics and Genomics (ACMG) guidelines (4) and recommendations published by Ellard et al (5). Only variants classified as likely pathogenic (class 4) or pathogenic (class 5) were included in the study. Copy number variant (CNV) analysis was performed using ExomeDepth according to the methodology described by Parrish et al. (14). The estimated sensitivity for CNV detection was >95%. Pathogenic or likely pathogenic CNVs were confirmed by multiplex ligation-dependent probe assay (SALSA MLPA P241 MODY kit, MRC-Holland). *HNF1B* analysis was performed by Sanger sequencing and MLPA dosage analysis as described previously (10). The m.3243A>G mutation was confirmed by TaqMan real-time PCR according to the method described previously (9). Heteroplasmy was measured in peripheral blood and levels of m.3243A>G above 3% were considered diagnostic for mitochondrial diabetes (12). The m.3243A>G heteroplasmy level was calculated as the number of sequence reads containing the mutation expressed as a percentage of the total number of reads aligned to the m.3243 locus. Heteroplasmy level was not assessed in any other tissues due to the lower prior likelihood of MIDD in our study cohort. The blood heteroplasmy level was corrected for age using a published method (15).

### Statistical analyses

Data were analyzed using STATA 16 (StataCorp, Texas, USA). Mann-Whitney U and Fisher Exact tests were used to compare continuous and categorical variables respectively.

## Results

### Characteristics of the cohort

The clinical characteristics of the 1280 participants who were referred from routine clinical care with suspected MODY are presented in Supplementary Table 2. The median age of diabetes diagnosis was 20 years (IQR 14-29), median diabetes duration was 3 years (IQR 1-12) and median BMI was 25.7 (IQR 22.4-30.0). Half of the cohort were non-insulin treated (627/1280, 49%) and 68% (873/1280) had a parent affected with diabetes. None of the patients were clinically suspected of having a mutation in a syndromic diabetes gene.

### Mutations in syndromic diabetes genes accounted for 19% of all monogenic cases in patients with suspected MODY

**Figure 1:**
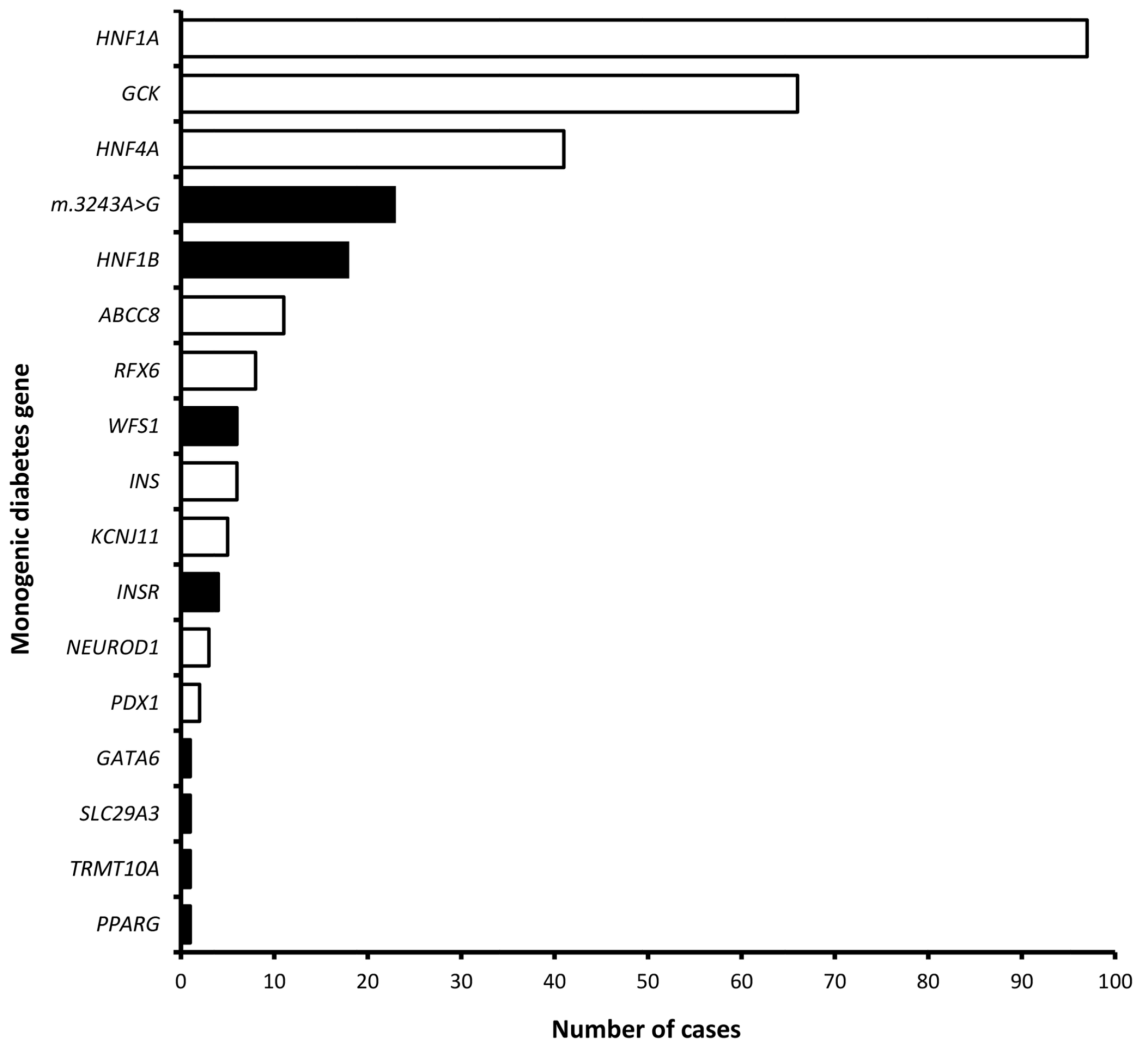
Bar chart showing number of cases for each monogenic diabetes gene. Filled bars are syndromic monogenic diabetes genes and open bars are non-syndromic monogenic diabetes genes.

We confirmed monogenic diabetes in 23% (297/1280) of cases (Fig. 1, Supplementary Tables 3 and 4).

Mutations in syndromic diabetes genes accounted for 19% (56/297, 95%CI 15-24%) of monogenic cases (Fig. 1). The mitochondrial mutation m.3243A>G was the most common syndromic subtype accounting for 43% (24/56) of all syndromic cases followed by mutations in *HNF1B* (n=18/56, 32%, 14 with a gene deletion and 4 with an SNV). These were the 4^th^ and 5^th^ most common monogenic causes overall. Mutations in 6 other genes were responsible for the remaining syndromic cases (14/56, 25%) (Fig. 1, Supplementary Table 3).

### Clinical features of patients with mutations in syndromic diabetes genes overlapped with patients with mutations in non-syndromic genes

We next compared the clinical features of patients with a syndromic diabetes gene mutation to patients with a mutation in a non-syndromic gene (Table 1). Both groups had similar age at diagnosis of diabetes, BMI and HbA1c. Patients with a mutation in a syndromic gene were more likely to be insulin treated (71% vs 39%, *P*<0.001) and less likely to have a parent affected with diabetes (53% vs 76%, *P*=0.001). They were more likely to have extra-pancreatic clinical features (23% vs 6%, *P*<0.001) but no patients had a constellation of features that pointed to a specific genetic syndrome.

### m.3243A>G cases identified in a suspected MODY cohort have atypical presentations

**Figure 2:**
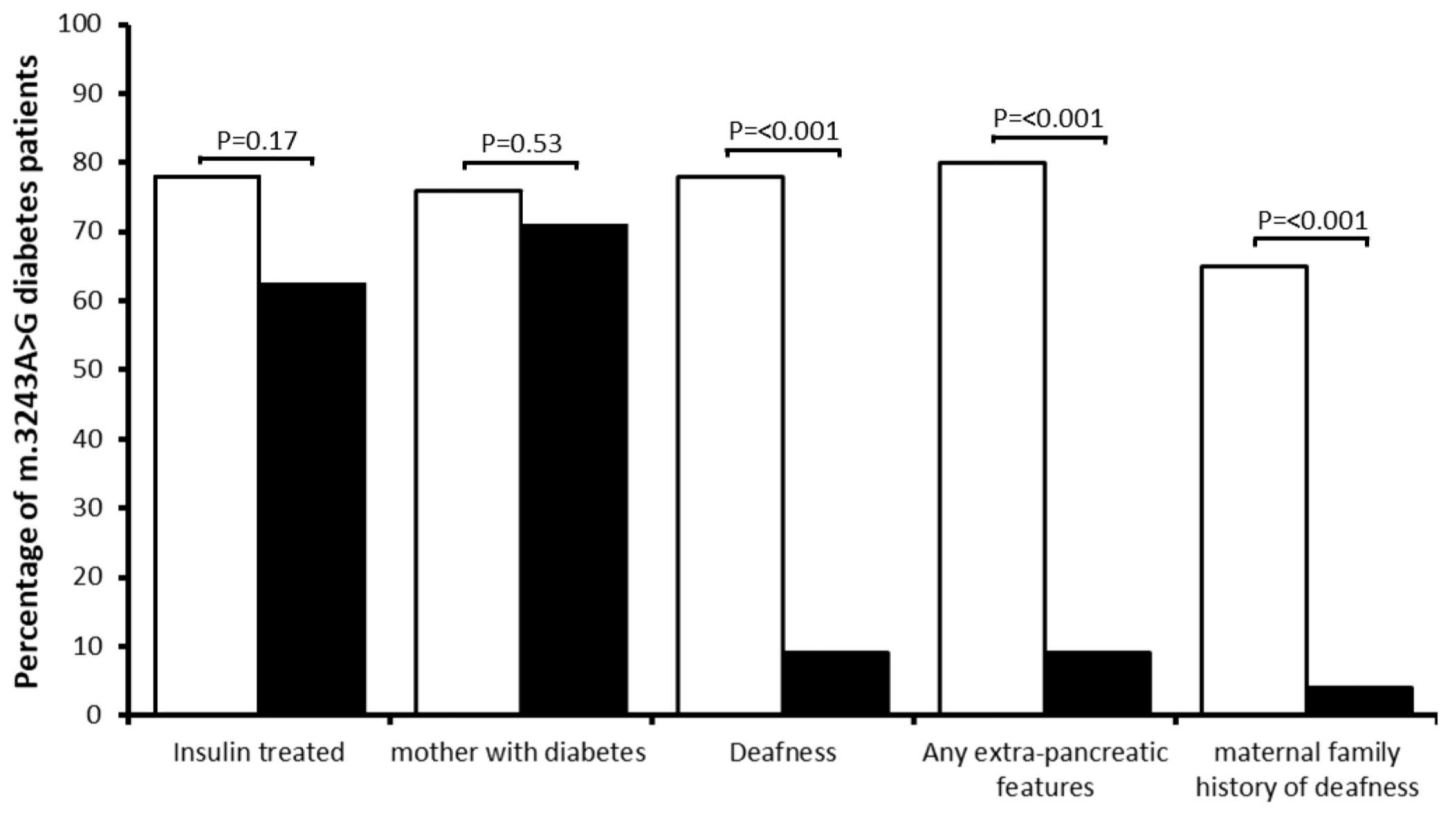
Comparison of clinical features in patients with m.3243A>G diabetes diagnosed by unselected testing using tNGS and by clinically suspected testing using a TaqMan genotyping assay undertaken as requested by the referring clinician. Filled bars are patients with diabetes and the m.3243A>G mutation identified by targeted tNGS in a suspected MODY cohort and unfilled bars are patients with m.3243A>G identified when clinically suspected of having MIDD.

We compared the clinical features of m.3243A>G cases identified in our suspected MODY cohort to patients that were diagnosed with m.3243A>G when clinically suspected of having MIDD. We found no significant difference in sex, age of diabetes diagnosis, BMI, HbA1c, diabetes treatment and maternal history of diabetes (Fig. 2b, Supplementary Table 5). Clinician reported deafness and maternal history of deafness (cardinal features of m.3243A>G) were significantly less common compared to the clinically suspected group (9% vs 78%, *P*<0.001 for deafness and 4% vs 65%, *P*<0.001 for maternal deafness). No patient in the suspected MODY cohort had any other extra-pancreatic features associated with m.3243A>G mutation. The median blood heteroplasmy level in the 24 patients with m.3243A>G detected by tNGS was 24.4% (IQR 18.1-33.8) and the median age corrected blood heteroplasmy was 79.6% (IQR 60.7-92.8). The age-adjusted blood heteroplasmy level was not associated with age at diagnosis of diabetes (beta -0.08 (95% CI–0.24, 0.75) P=0.28 (supplementary figure 1)) and maternal diabetes status (median 85.5% [IQR 73.7-125] with maternal diabetes vs 77.6% [IQR 53.8-90] without maternal diabetes, P=0.13). The two people with deafness had a marginally higher age-adjusted blood heteroplasmy level compared to those without deafness (94.9% and 99.4%] vs median 78.3% [IQR, 58.6-90], P=0.11).

### Cases with an *HNF1B* mutation in a suspected MODY cohort had atypical presentation

**Figure 3:**
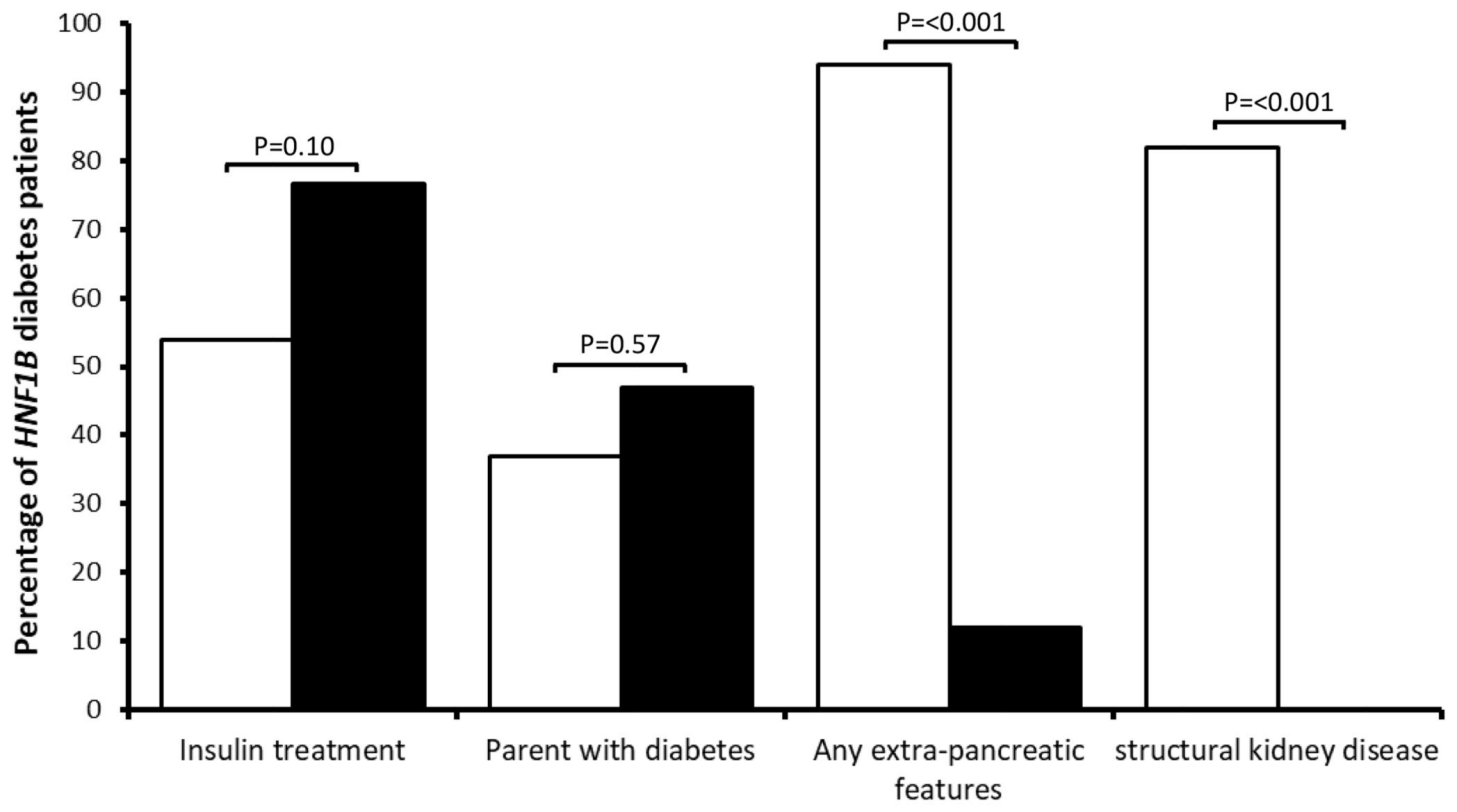
Comparison of clinical features in patients with *HNF1B* diabetes diagnosed by unselected testing using tNGS and by clinically suspected testing using Sanger sequencing and MLPA analysis undertaken as requested by the referring clinician. Filled bars are patients with an *HNF1B* mutation identified by targeted NGS in a suspected MODY cohort and non-filled bars are patients with an *HNF1B* mutation identified when clinically suspected of having *HNF1B*-related disease.

We observed no significant difference in age of diabetes diagnosis, BMI, HbA1c, diabetes treatment or parental diabetes in patients with an *HNF1B* mutation in our suspected MODY cohort compared to cases identified when *HNF1B* diabetes was clinically suspected (Supplementary Table 6). Extra-pancreatic features were less common in patients diagnosed in our cohort compared to those by clinically suspected testing (11% vs 94%, *P*<0.001) (Fig. 3 and Supplementary Table 6). Structural kidney disease (renal cysts, dysplasia and hypoplasia/agenesis that are the cardinal features of *HNF1B* disease) was not reported in any of the patients diagnosed by unselected genetic testing. Non-kidney features were reported in two patients with a whole gene deletion; one patient had autism and the other had a rudimentary uterus and hypoplastic ovaries. The lack of extra-pancreatic features was still observed when analysis was restricted to patients with a whole-gene deletion (Supplementary Table 7).

### Genetic diagnosis led to identification of extra-pancreatic features in patients with mutations in syndromic diabetes genes

Mutations in syndromic diabetes genes other than m.3243A>G and *HNF1B* were identified in 14 patients but none had clinical features at referral that were suggestive of having mutations in any of these genes (Supplementary Table 8). We re-contacted the clinicians of these patients and obtained follow-up information on 12 (five *WFS1*, four *INSR*, one each of *GATA6, TRMT10A* and *PPARG*) (Supplementary Table 8). In 7/12 (58%) patients there were unreported clinical features that would have supported the final genetic diagnosis but there were no known features present in 5/12 (42%) patients at time of genetic testing. With further investigation/follow-up after the genetic diagnosis was made all patients had features consistent with their syndrome except the patient with *GATA6* diabetes. We also re-contacted the clinicians of patients with m.3243A>G and *HNF1B* diabetes identified by tNGS and obtained follow-up information on 15 cases (5/18 cases with *HNF1B* and 10/24 cases with m.3243A>G). We found that only 2 cases (13%) had characteristic syndromic features which were not reported by the clinician at the time of genetic testing. One clinician failed to report renal cysts for an *HNF1B* diabetes patient, and deafness in another patient with the m.3243A>G mutation. At median follow-up of 5.4 years (range 3.8-7 years), three of the four remaining *HNF1B* patients were found to have renal cysts whereas only one of the remaining nine patients with m.3243A>G developed deafness. In total, after follow-up, 11/56 (19.6%) patients had features which would have predicted the presence of the syndromic gene mutation. Even if we remove these patients from the study, mutations in syndromic genes still accounted for 16% (46/287, 95%CI 12-20%) of all monogenic cases.

## Discussion

Our study in a real-world setting strongly supports routine testing of syndromic diabetes genes in patients with suspected MODY. We showed that 1 in 5 patients with suspected MODY had a mutation in a syndromic diabetes gene and lacked typical features. It is the overlapping diabetes features with MODY that results in the referral of these patients for genetic testing. Their diagnosis would be missed using the current strategy that restricts testing of syndromic genes to those patients with characteristic clinical features.

The m.3243A>G mutation is the 4^th^ most common cause of monogenic diabetes (8% of all monogenic cases) after mutations in *GCK*, *HNF1A* and *HNF4A* in patients with suspected MODY. There have been numerous studies of genetic testing in clinically suspected MODY cohorts but only one small study of 109 patients from Korea included m.3243A>G (16). This lack of m.3243A>G testing is also seen in the NCBI Genetic Testing Registry where none of the 26 gene panels for MODY included m.3243A>G testing (8). All patients with m.3243A>G in our suspected MODY cohort lacked typical features of MIDD; only two patients had deafness but reportedly due to drug toxicity and ear infection, and our follow-up of 10 cases identified only one additional patient with deafness. Even if we remove these three cases from our calculation, m.3243A>G remains the most common syndromic subtype accounting for 39% (21/53) of syndromic cases and 7% (21/294) of all monogenic cases. The low prevalence of deafness in our m.3243A>G patients suggests that significant variable expressivity is the mostly likely reason for the non-syndromic appearance of MIDD patients and not the lack of reporting by clinicians. This data is consistent with previous reports of significant variable expressivity in MIDD (17).

Previous studies have suggested that heteroplasmy levels explain up to 27% of the variation in disease burden of m.3243A>G (15). We saw no association of heteroplasmy with age of diabetes diagnosis, maternal diabetes status and maternal deafness status. However, the small sample size of our study prevents firm conclusions from being made. Most patients had an intermediate level of heteroplasmy suggesting that the lack of severe hearing loss is not due to a low blood heteroplasmy level. Further studies are needed to compare heteroplasmy levels of patients identified from the MODY cohort to patients diagnosed due to a clinical suspicion of MIDD.

*HNF1B* mutations were also common in patients with suspected MODY but lacking renal features suggestive of *HNF1B* disease. This finding was seen in a previous large study but at a lower frequency (10%) (18). *HNF1B* is also included in 24/26 MODY gene panels from NCBI gene registry highlighting the awareness of testing *HNF1B* in suspected MODY patients. 78% of patients in our study with *HNF1B* diabetes had a large partial (one or more exons) or whole gene deletion. Conventional variant calling performed by GATK haplotype caller does not detect these large deletions, and they can only be detected by performing CNV analysis as a part of the NGS bioinformatics pipeline. In our whole study, 16/297 (5.4%) of patients had monogenic diabetes due to either a partial or whole gene deletion (14 *HNF1B*, 1 *HNF1A* and 1 *HNF4A*) (Supplementary Table 4). CNV analysis is performed on already available data generated by tNGS with minimal cost implications and has the benefit of additional genetic diagnosis. CNV analysis should therefore be performed as part of NGS testing for MODY. However, this is currently rarely performed in published studies of MODY testing using NGS (9; 19).

We also identified 14 cases with mutations in syndromic monogenic diabetes genes other than m.3243A>G and *HNF1B*. In over half of cases the characteristic features were present at referral but the clinician did not associate them with the cause of the diabetes and thus did not report at genetic testing. The lack of any specific features in 40% of patients was due to variable expressivity in these genes as reported previously (7; 11; 20; 21). Our study also shows that the simple clinical features which may suggest a monogenic cause of the diabetes are not reported at least in some cases. This highlights the need for continuing professional education about monogenic diabetes for clinicians that see only a handful of monogenic cases in their careers due to the rarity of the disease.

Including syndromic genes on MODY panels has a number of benefits. It removes the need for clinicians to have detailed knowledge of all monogenic diabetes syndromes and focuses on identifying patients with a clinical suspicion of monogenic diabetes using tools that are independent of aetiology (e.g. C-peptide, islet auto-antibodies and type 1 diabetes genetic risk score) (3; 22). A diagnosis of syndromic monogenic diabetes provides prognostic information and may prompts clinicians to screen for the presence of additional features, providing an opportunity to treat early in the disease process (e.g. screening for renal cysts and kidney function in *HNF1B* diabetes or cardiomyopathy in m.3243A>G diabetes). The genetic diagnosis may also explains the presence of additional features and may prevents unnecessary investigations to explain these features (e.g. raised liver enzymes with *HNF1B* diabetes or myopathy with m.3243A>G diabetes). It is recommended that patients with genetic syndromes are reviewed by clinical genetic services. Support from clinical genetics services and specialist clinics are needed, particularly when an unexpected diagnosis of a genetic syndrome is made, to prevent significant anxiety and provide holistic management. This strategy also requires extra caution in interpreting novel variants in syndromic genes identified in patients lacking typical features of the syndrome (23).

77% of patients did not receive a genetic diagnosis. MODY has overlapping features with young-onset type 1 and type 2 diabetes, and no single criterion can identify all MODY patients (24). In the UK, all children with negative GAD, IA2 and ZnT8 islet autoantibodies and detectable C-peptide and adults diagnosed <35 years with >20% prior probability of MODY are recommended to have genetic testing (25) with the aim of identifying the majority of patients with MODY at the expense of a lower positive predictive value due to the testing of polygenic atypical type 1 and type 2 diabetes cases. The lack of genetic diagnosis in 77% of cases is therefore more likely due to the inclusion of atypical type 1 and type 2 diabetes, with a minority of cases due to yet unknown novel monogenic diabetes or non-coding mutations in known genes not detected by our assay. We did not include *BLK, KLF11* and *PAX4* in our gene panel due to lack of strong genetic evidence supporting the gene-disease association for MODY (26). *APPL1* is a very rare putative MODY gene that was only tested in 36% of our tNGS cohort and therefore not included in the study. This lower prior likelihood of monogenic diabetes has important implications for assessment of pathogenicity of a detected novel variant. This is particularly important for genes that cause syndromic diabetes in our study as the phenotype is used as evidence when classifying variant pathogenicity (27). Novel missense variants in cohorts of patients with a low prior probability of monogenic diabetes are more likely to be benign or have uncertain clinical significance, particularly when patients lack the typical features of the syndrome (28).

A limitation of our study is the lack of long-term clinical follow-up of all m.3243A>G and *HNF1B* patients to determine whether they are truly atypical cases. However, our limited follow-up on one third of the patients and the specific request for renal disease and deafness status on our referral form suggests that it is likely that these patients are not severely affected. A further clinical study with longer follow-up duration is needed to assess the stability of the non-syndromic appearance.

In conclusion, mutations in syndromic monogenic diabetes genes are common in patients with suspected MODY in routine clinical practice. We strongly recommend including syndromic diabetes genes in gene panel tests for MODY to enable early diagnosis of atypical presentations and clinical benefits for diagnosed patients.

## Supplementary Material

Supplementary tables 1 to 8 and Supplementary figure 1

## Figures and Tables

**Table 1:** Characteristics of patients with mutations in syndromic diabetes genes and non-syndromic diabetes genes. Data is in the format median, (IQR), total for continuous variables, and n/total (%) for categorical variables.

Characteristic	Patients with mutations in syndromic monogenic diabetes genes	Patients with mutations in non-syndromic monogenic diabetes genes	P
N	56	241	
Age at diagnosis of diabetes (y)	20 (13.5-29), 56	17 (13-25), 241	0.09
Diabetes duration (y)	4 (1-8.5), 56	3 (0.5-14), 241	0.89
Female	37 (66%)	145 (60%)	0.44
BMI (kg/m^2^)	22.0 (20.0-26.9), 49	23.7 (21.2-27.6), 197	0.05
Extra-pancreatic features	13 (23%)	15 (6%)	<0.001
Parent with diabetes	30 (53%)	184 (76%)	0.001
Ethnicity (non-white)	13 (23%)	38 (16%)	0.23
HbA1c (%)	7.3 (6.5-9.5), 41	7 (6.3-8.4), 199	0.20
HbA1c (mmol/mol)	56 (48-80)	53 (45-68)	0.20
Insulin treated	40 (71%)	95 (39%)	<0.001
Insulin alone	33	73	
Insulin with Oral Hypoglycaemic Drugs	7	22	

## Data Availability

All mutations identified in the study are provided in online supplementary materials file. The clinical data generated and/or analyzed as part of this study is not publicly available due to patient confidentiality but is available from the corresponding authors on reasonable request. Clinical information on individual patients with specific monogenic diabetes mutations is available on request in order to assist other laboratories and clinicians with variant interpretation and genetic diagnosis of their patients.

## References

[1] Pearson ER, Starkey BJ, Powell RJ, Gribble FM, Clark PM, Hattersley AT (2003). Genetic cause of hyperglycaemia and response to treatment in diabetes. Lancet.

[2] Stride A, Shields B, Gill-Carey O, Chakera AJ, Colclough K, Ellard S, Hattersley AT (2014). Cross-sectional and longitudinal studies suggest pharmacological treatment used in patients with glucokinase mutations does not alter glycaemia. Diabetologia.

[3] Shields BM, Shepherd M, Hudson M, McDonald TJ, Colclough K, Peters J, Knight B, Hyde C, Ellard S, Pearson ER, Hattersley AT (2017). Population-Based Assessment of a Biomarker-Based Screening Pathway to Aid Diagnosis of Monogenic Diabetes in Young-Onset Patients. Diabetes Care.

[4] Clissold RL, Hamilton AJ, Hattersley AT, Ellard S, Bingham C (2015). HNF1B-associated renal and extrarenal disease-an expanding clinical spectrum. Nat Rev Nephrol.

[5] Murphy R, Turnbull DM, Walker M, Hattersley AT (2008). Clinical features, diagnosis and management of maternally inherited diabetes and deafness (MIDD) associated with the 3243A>G mitochondrial point mutation. Diabet Med.

[6] Stone SI, Abreu D, McGill JB, Urano F (2021). Monogenic and syndromic diabetes due to endoplasmic reticulum stress. J Diabetes Complications.

[7] Yaghootkar H, Abbasi F, Ghaemi N, Rabbani A, Wakeling MN, Eshraghi P, Enayati S, Vakili S, Heidari S, Patel K, Sayarifard F (2019). Type 1 diabetes genetic risk score discriminates between monogenic and Type 1 diabetes in children diagnosed at the age of <5 years in the Iranian population. Diabet Med.

[8] (2021). GTR: Genetic Testing Registry.

[9] Bansal V, Gassenhuber J, Phillips T, Oliveira G, Harbaugh R, Villarasa N, Topol EJ, Seufferlein T, Boehm BO (2017). Spectrum of mutations in monogenic diabetes genes identified from high-throughput DNA sequencing of 6888 individuals. BMC Med.

[10] Dubois-Laforgue D, Cornu E, Saint-Martin C, Coste J, Bellanne-Chantelot C, Timsit J, Monogenic Diabetes Study Group of the Societe Francophone du D (2017). Diabetes, Associated Clinical Spectrum, Long-term Prognosis, and Genotype/Phenotype Correlations in 201 Adult Patients With Hepatocyte Nuclear Factor 1B (HNF1B) Molecular Defects. Diabetes Care.

[11] Gonzaga-Jauregui C, Ge W, Staples J, Van Hout C, Yadav A, Colonie R, Leader JB, Kirchner HL, Murray MF, Reid JG, Carey DJ (2020). Clinical and Molecular Prevalence of Lipodystrophy in an Unascertained Large Clinical Care Cohort. Diabetes.

[12] Pickett SJ, Grady JP, Ng YS, Gorman GS, Schaefer AM, Wilson IJ, Cordell HJ, Turnbull DM, Taylor RW, McFarland R (2018). Phenotypic heterogeneity in m.3243A>G mitochondrial disease: The role of nuclear factors. Ann Clin Transl Neurol.

[13] Ellard S, Lango Allen H, De Franco E, Flanagan SE, Hysenaj G, Colclough K, Houghton JA, Shepherd M, Hattersley AT, Weedon MN, Caswell R (2013). Improved genetic testing for monogenic diabetes using targeted next-generation sequencing. Diabetologia.

[14] (2017). An enhanced method for targeted next generation sequencing copy number variant detection using ExomeDepth.

[15] Grady JP, Pickett SJ, Ng YS, Alston CL, Blakely EL, Hardy SA, Feeney CL, Bright AA, Schaefer AM, Gorman GS, McNally RJ (2018). mtDNA heteroplasmy level and copy number indicate disease burden in m.3243A>G mitochondrial disease. EMBO Mol Med.

[16] Park SS, Jang SS, Ahn CH, Kim JH, Jung HS, Cho YM, Lee YA, Shin CH, Chae JH, Kim JH, Choi SH (2019). Identifying Pathogenic Variants of Monogenic Diabetes Using Targeted Panel Sequencing in an East Asian Population. J Clin Endocrinol Metab.

[17] Mancuso M, Orsucci D, Angelini C, Bertini E, Carelli V, Comi GP, Donati A, Minetti C, Moggio M, Mongini T, Servidei S (2014). The m.3243A>G mitochondrial DNA mutation and related phenotypes. A matter of gender? J Neurol.

[18] Donath X, Saint-Martin C, Dubois-Laforgue D, Rajasingham R, Mifsud F, Ciangura C, Timsit J, Bellanne-Chantelot C, Monogenic Diabetes Study Group of the Societe Francophone du D (2019). Nextgeneration sequencing identifies monogenic diabetes in 16% of patients with late adolescence/adult-onset diabetes selected on a clinical basis: a cross-sectional analysis. BMC Med.

[19] Johansson BB, Irgens HU, Molnes J, Sztromwasser P, Aukrust I, Juliusson PB, Sovik O, Levy S, Skrivarhaug T, Joner G, Molven A (2017). Targeted next-generation sequencing reveals MODY in up to 6.5% of antibody-negative diabetes cases listed in the Norwegian Childhood Diabetes Registry. Diabetologia.

[20] Aghababaie AS, Ford-Adams M, Buchanan CR, Arya VB, Colclough K, Kapoor RR (2020). A novel heterozygous mutation in the insulin receptor gene presenting with type A severe insulin resistance syndrome. J Pediatr Endocrinol Metab.

[21] De Franco E, Shaw-Smith C, Flanagan SE, Shepherd MH, Hattersley AT, Ellard S, International NDMC (2013). GATA6 mutations cause a broad phenotypic spectrum of diabetes from pancreatic agenesis to adult-onset diabetes without exocrine insufficiency. Diabetes.

[22] Patel KA, Weedon MN, Shields BM, Pearson ER, Hattersley AT, McDonald TJ, team Us (2019). Zinc Transporter 8 Autoantibodies (ZnT8A) and a Type 1 Diabetes Genetic Risk Score Can Exclude Individuals With Type 1 Diabetes From Inappropriate Genetic Testing for Monogenic Diabetes. Diabetes Care.

[23] Ellard S, Colclough K, Patel KA, Hattersley AT (2020). Prediction algorithms: pitfalls in interpreting genetic variants of autosomal dominant monogenic diabetes. J Clin Invest.

[24] Shields BM, McDonald TJ, Ellard S, Campbell MJ, Hyde C, Hattersley AT (2012). The development and validation of a clinical prediction model to determine the probability of MODY in patients with young-onset diabetes. Diabetologia.

[25] (2021). Tests For Diabetes Subtypes.

[26] (2021). Evaluation of evidence for pathogenicity demonstrates that BLK, KLF11 and PAX4 should not be included in diagnostic testing for MODY.

[27] Richards S, Aziz N, Bale S, Bick D, Das S, Gastier-Foster J, Grody WW, Hegde M, Lyon E, Spector E, Voelkerding K (2015). Standards and guidelines for the interpretation of sequence variants: a joint consensus recommendation of the American College of Medical Genetics and Genomics and the Association for Molecular Pathology. Genet Med.

[28] Shields B, Colclough K (2017). Towards a systematic nationwide screening strategy for MODY. Diabetologia.

