# Supplementary material for: Syndromic monogenic diabetes genes should be tested in patients with a clinical suspicion of MODY
Source: Diabetes. Author manuscript; Available in PMC 2022 Mar 1. (PMC7612420; doi:10.2337/db21-0517)
Supplement: Supplementary tables 1 to 8 and Supplementary figure 1 [file EMS140031-supplement-Supplementary_tables_1_to_8_and_Supplementary_figure_1.pdf]

Supplementary Material

Supplementary table 1: List of monogenic diabetes genes and mitochondrial DNA mutation m.3243A>G analysed in this study. *Common non-syndromic refers to the three most common causes of isolated, non-syndromic monogenic diabetes (MODY) diagnosed outside of the neonatal period (*GCK*, *HNF1A* and *HNF4A*). Non-syndromic specifically relates to heterozygous mutations causing isolated monogenic diabetes, and excludes any syndromes associated with biallelic mutations in the same gene (e.g. heterozygous mutations in *RFX6* cause MODY with reduced penetrance, whereas biallelic mutations cause Mitchell-Riley Syndrome).

Gene (OMIM)	Category*	Phenotype	OMIM	Inheritance	References
ABCC8 600509	Other non-syndromic	MODY	610374	Dominant	Bowman *et al* 2012 Diabetologia 55: 123-127<br>Riveline *et al* 2012 Diabetes Care 35: 248-251
CEL 114840	Syndromic	MODY and pancreatic exocrine dysfunction	609812	Dominant	Raeder *et al* 2006 Nat Genet 38: 54-62<br>Torsvik *et al* 2010 Hum Genet 127: 55-64<br>Raeder *et al* 2013 PLoS One 8: e60229
CISD2 611507	Syndromic	Wolfram Syndrome 2 (diabetes mellitus, hearing loss, optic atrophy and defective platelet aggregation).	604928	Recessive	Amr *et al* 2007 Amr J Hum Genet 81: 673-683
GATA4 600576	Syndromic	Permanent neonatal diabetes with pancreatic agenesis and congenital heart defects	Not yet assigned	Dominant (often *de novo*)	D'Amato *et al* 2010 Diabet Med 27: 1195-1200
GATA6 601656	Syndromic	Permanent neonatal diabetes with pancreatic agenesis and congenital heart defects	600001	Dominant (often *de novo*)	Lango Allen *et al* 2011 Nat Genet 44: 20-22<br>De Franco *et al* 2013 Diabetes 62: 993-997

GCK 138079	Common non-syndromic	MODY	125851	Dominant	Vionnet *et al* 1992 Nature 356: 721-722<br>Velho *et al* 1997 Diabetologia 40: 217-224<br>Osbak *et al* 2009 Hum Mutat 30: 1512-1526
HNF1A 142410	Common non-syndromic	MODY	600496	Dominant	Yamagata *et al* 1996 Nature 384: 455-458<br>Frayling *et al* 1997 Diabetes 46: 720-725<br>Colclough *et al* 2013 Hum Mutat 34: 669-685
HNF1B 189907	Syndromic	Renal Cysts and Diabetes syndrome (RCAD)	137920	Dominant (often *de novo*)	Horikawa *et al* 1997 Nat Genet 17: 384-385<br>Yorifuji *et al* 2004 J Clin Endocrinol Metab 89: 2905-2908<br>Edghill *et al* 2006 J Med Genet 43: 84-90<br>Bellanne-Chantelot *et al* 2005 Diabetes 54: 3126-3132
HNF4A 600281	Common non-syndromic	MODY	125850	Dominant	Yamagata *et al* 1996 Nature 384: 458-460<br>Bulman *et al* 1997 Diabetologia 40: 859-862<br>Colclough *et al* 2013 Hum Mutat 34: 669-685
INS 176730	Other non-syndromic	MODY	613370	Dominant	Edghill *et al* 2008 Diabetes 57: 1034-1042<br>Molven *et al* 2008 Diabetes 57: 1131-1135
INSR 147670	Syndromic	Severe insulin resistance	610549	Dominant	Odawara *et al* 1989 Science 245: 66-68
KCNJ11 600937	Other non-syndromic	MODY	616329	Dominant	Yorifuji *et al* 2005 J Clin Endocrinol Metab 90: 3174-3178<br>Bonnefond *et al* 2012 PLoS One 7: e37423
LMNA 150330	Syndromic	Familial Partial Lipodystrophy (FPLD2) and insulin resistance	151660	Dominant	Cao *et al* 2000 Hum Mol Genet 1: 109-112<br>Shackleton *et al* 2000 Nat Genet 24: 153-156<br>Speckman *et al* 2000 Am J Hum Genet 66: 1192-1198

MTTL1 m.3243A>G 590050	Syndromic	Maternally inherited diabetes and deafness (MIDD)	520000	Mitochondrial	Van den Ouweland *et al* 1992 Nat Genet 1: 368-371<br>Murphy *et al* 2008 Diabet Med 25: 383-399
NEUROD1 601724	Other non-syndromic	MODY	606394	Dominant	Malecki *et al* 1999 Nat Genet 23: 323-328<br>Kristinsson *et al* 2001 Diabetologia 44: 2098-2103
PAX6 607108	Syndromic	Aniridia and impaired glucose tolerance	106210	Dominant	Yasuda *et al* 2002 Diabetes 51: 224-230<br>Nishi *et al* 2005 Diabet Med 22: 641-644<br>Osawa *et al* 2015 J Diabetes Investig 6: 105-106
PCBD1 126090	Syndromic	Diabetes and hyperphenylalaninaemia	264070	Recessive	Simaite *et al* 2014 Diabetes 63: 3557-3564<br>Ferre *et al* 2014 J Am Soc Nephrol 25: 574-586
PDX1 600733	Other non-syndromic	MODY	606392	Dominant	Stoffers *et al* 1997 Nat Genet 17: 138-139
PLIN1 170290	Syndromic	Familial Partial Lipodystrophy (FPLD4) and insulin resistance	613877	Dominant	Gandotra *et al* 2011 N Engl J Med 364: 740-748
POLD1 174761	Syndromic	Mandibular hypoplasia, deafness, progeroid features, and lipodystrophy (MDPL) syndrome	615381	Dominant (*de novo*)	Weedon *et al* 2013 Mat Genet 45: 947-950
PPARG 601487	Syndromic	Familial Partial Lipodystrophy (FPLD3) and insulin resistance	604367	Dominant	Agarwal *et al* 2002 J Clin Endocrinol Metab 1: 408-411<br>Barroso *et al* 1999 Nature 402: 880-883
RFX6 612659	Other non-syndromic	MODY with reduced penetrance	Not yet assigned	Dominant	Patel *et al* 2017 Nat Commun 8: 888

SLC29A3 612373	Syndromic	H syndrome & PHID syndrome	602782	Recessive	Cliffe *et al* 2009 Hum Molec Genet 18: 2257-2265<br>Molho-Pessach *et al* 2008 Am J Hum Genet 83: 529-534
TRMT10A 616013	Syndromic	Juvenile-onset diabetes with microcephaly, epilepsy and intellectual disability	616033	Recessive	Igoillo-Esteve *et al* 2013 PLoS Genet 9: e1003888
WFS1 606201	Syndromic	Wolfram syndrome (Diabetes insipidus, diabetes mellitus, optic atrophy and deafness, DIDMOAD)	222300	Recessive	Inoue *et al* 1998 Nat Genet 20: 143-148<br>Strom *et al* 1998 Hum Mol Genet 7: 2021-2028
ZBTB20 606025	Syndromic	Primrose syndrome	259050	Dominant (*de novo*)	Cordeddu *et al* 2014 Nat Genet 46: 815-817
ZFP57 612192	Syndromic	Transient neonatal diabetes	601410	Recessive	Mackay *et al* 2008 Nat Genet 40: 949-951

Supplementary Table 2: Clinical characteristics of the whole cohort. Data is in the format median, (IQR), n for continuous variables and n (%) for categorical variables.

**Characteristic**	**All probands (n=1280)**
Age at diagnosis (y)	20 (14-29), 1280
Duration (y)	3 (1-12), 1280
Female	724 (57%)
BMI (kg/m^2)	25.7 (22.4-30.0), 1058
Extra-pancreatic features	151 (12%)
Parent with diabetes	873 (68%)
Ethnicity (non-white)	334 (26%)
HbA1c (%)	7.6 (6.5-9.5), 976
HbA1c (mmol/mol)	60 (48-80)
Insulin alone or with Oral Hypoglycaemic Drugs	653 (51%)

Supplementary Table 3: Genetic causes of monogenic diabetes in our cohort. *Common non-syndromic refers to the three most common causes of isolated, non-syndromic monogenic diabetes (MODY) diagnosed outside of the neonatal period (*GCK*, *HNF1A* and *HNF4A*). Non-syndromic specifically relates to heterozygous mutations causing isolated monogenic diabetes, and excludes any syndromes associated with biallelic mutations in the same gene (e.g. heterozygous mutations in *RFX6* cause MODY with reduced penetrance, whereas biallelic mutations cause Mitchell-Riley Syndrome).

Gene	Category of genetic aetiologies*	Number of probands	Proportion of all monogenic diabetes cases
HNF1A	Common non-syndromic	98	33%
GCK	Common non-syndromic	66	22%
HNF4A	Common non-syndromic	42	14%
m.3243A>G	Syndromic	24	8%
HNF1B	Syndromic	18	6%
ABCC8	Other non-syndromic	11	4%
RFX6	Other non-syndromic	8	3%
WFS1	Syndromic	6	2%
INS	Other non-syndromic	6	2%
KCNJ11	Other non-syndromic	5	2%
INSR	Syndromic	4	1%
NEUROD1	Other non-syndromic	3	1%
PDX1	Other non-syndromic	2	<1%
GATA6	Syndromic	1	<1%
SLC29A3	Syndromic	1	<1%
TRMT10A	Syndromic	1	<1%

PPARG	Syndromic	1	<1%
	**Total**	**297**	

Supplementary Table 4: List of pathogenic and likely pathogenic variants identified in this study. All variants described using HGVS nomenclature (https://varnomen.hgvs.org/) based on the following NCBI Reference Sequences (RefSeq): *HNF1A* NM_000545.8, *HNF4A* NM_175914.4, *GCK* NM_000162.5, *ABCC8* NM_001287174.2, *INS* NM_001185098.2, *KCNJ11* NM_000525.4, *NEUROD1* NM_002500.5, *PDX1* NM_000209.4, *RFX6* NM_173560.4, *HNF1B* NM_000458.4, *GATA6* NM_005257.5, *INSR* NM_000208.4, *PPARG* NM_015869.4, *SLC29A3* NM_018344.5, *TRMT10A* NM_152292.5, *WFS1* NM_006005.3 and *MT-TL1* NC_012920.1.

**Gene**	**DNA change**	**Protein Change**	**Zygosity**	**Variant type**	**Classification**	**Number of probands**
HNF1A	c.(?_-1)_(*1_?)del	p.0?	Heterozygous	Whole gene deletion	Pathogenic	1
HNF1A	c.-258A>G	p.0?	Heterozygous	Regulatory	Likely Pathogenic	1
HNF1A	c.25C>T	p.(Gln9*)	Heterozygous	Nonsense	Pathogenic	1
HNF1A	c.34C>G	p.(Leu12Val)	Heterozygous	Missense	Likely Pathogenic	1
HNF1A	c.44C>T	p.(Ala15Val)	Heterozygous	Missense	Likely Pathogenic	1
HNF1A	c.46C>G	p.(Leu16Val)	Heterozygous	Missense	Likely Pathogenic	1
HNF1A	c.56C>T	p.(Ser19Leu)	Heterozygous	Missense	Likely Pathogenic	1
HNF1A	c.58G>A	p.(Gly20Arg)	Heterozygous	Missense	Likely Pathogenic	1
HNF1A	c.85G>C	p.(Ala29Pro)	Heterozygous	Missense	Likely Pathogenic	1
HNF1A	c.197dup	p.(Thr67fs)	Heterozygous	Frameshift	Pathogenic	1
HNF1A	c.319C>G	p.(Leu107Val)	Heterozygous	Missense	Likely Pathogenic	1
HNF1A	c.325C>T	p.(Gln109*)	Heterozygous	Nonsense	Pathogenic	1
HNF1A	c.347C>T	p.(Ala116Val)	Heterozygous	Missense	Pathogenic	1
HNF1A	c.361T>C	p.(Ser121Pro)	Heterozygous	Missense	Likely Pathogenic	1
HNF1A	c.375G>T	p.(Gln125His)	Heterozygous	Missense	Likely Pathogenic	1
HNF1A	c.382A>G	p.(Ile128Val)	Heterozygous	Missense	Likely Pathogenic	1

HNF1A	c.391C>T	p.(Arg131Trp)	Heterozygous	Missense	Pathogenic	1
HNF1A	c.392G>A	p.(Arg131Gln)	Heterozygous	Missense	Pathogenic	2
HNF1A	c.476G>A	p.(Arg159Gln)	Heterozygous	Missense	Pathogenic	2
HNF1A	c.481G>A	p.(Ala161Thr)	Heterozygous	Missense	Likely Pathogenic	1
HNF1A	c.493T>C	p.(Trp165Arg)	Heterozygous	Missense	Likely Pathogenic	1
HNF1A	c.502C>T	p.(Arg168Cys)	Heterozygous	Missense	Likely Pathogenic	1
HNF1A	c.521C>T	p.(Ala174Val)	Heterozygous	Missense	Likely Pathogenic	1
HNF1A	c.526C>T	p.(Gln176*)	Heterozygous	Nonsense	Pathogenic	1
HNF1A	c.543del	p.(Gln182fs)	Heterozygous	Frameshift	Pathogenic	1
HNF1A	c.591G>T	p.(Lys197Asn)	Heterozygous	Missense	Likely Pathogenic	1
HNF1A	c.598C>T	p.(Arg200Trp)	Heterozygous	Missense	Pathogenic	2
HNF1A	c.599G>A	p.(Arg200Gln)	Heterozygous	Missense	Pathogenic	2
HNF1A	c.607C>T	p.(Arg203Cys)	Heterozygous	Missense	Pathogenic	1
HNF1A	c.608G>A	p.(Arg203His)	Heterozygous	Missense	Pathogenic	1
HNF1A	c.613A>C	p.(Lys205Gln)	Heterozygous	Missense	Likely Pathogenic	1
HNF1A	c.653A>G	p.(Tyr218Cys)	Heterozygous	Missense	Likely Pathogenic	1
HNF1A	c.673A>C	p.(Ser225Arg)	Heterozygous	Missense	Likely Pathogenic	1
HNF1A	c.685C>T	p.(Arg229*)	Heterozygous	Nonsense	Pathogenic	3
HNF1A	c.686G>A	p.(Arg229Gln)	Heterozygous	Missense	Pathogenic	1
HNF1A	c.718G>C	p.(Glu240Gln)	Heterozygous	Missense	Likely Pathogenic	1
HNF1A	c.779C>T	p.(Thr260Met)	Heterozygous	Missense	Pathogenic	1
HNF1A	c.809A>G	p.(Asn270Ser)	Heterozygous	Missense	Likely Pathogenic	2
HNF1A	c.811C>T	p.(Arg271Trp)	Heterozygous	Missense	Pathogenic	2
HNF1A	c.814C>T	p.(Arg272Cys)	Heterozygous	Missense	Pathogenic	1
HNF1A	c.815G>A	p.(Arg272His)	Heterozygous	Missense	Pathogenic	1
HNF1A	c.824A>C	p.(Glu275Ala)	Heterozygous	Missense	Likely Pathogenic	1
HNF1A	c.824_826del	p.(Glu275del)	Heterozygous	In-frame deletion	Pathogenic	2
HNF1A	c.827C>A	p.(Ala276Asp)	Heterozygous	Missense	Likely Pathogenic	2
HNF1A	c.872del	p.(Pro291fs)	Heterozygous	Frameshift	Pathogenic	5

HNF1A	c.872dup	p.(Gly292fs)	Heterozygous	Frameshift	Pathogenic	16
HNF1A	c.873del	p.(Pro293fs)	Heterozygous	Frameshift	Pathogenic	1
HNF1A	c.955+2T>C	p.?	Heterozygous	Aberrant Splicing	Pathogenic	1
HNF1A	c.1058_1059dup	p.(Thr354fs)	Heterozygous	Frameshift	Pathogenic	1
HNF1A	c.1107+2T>C	p.?	Heterozygous	Aberrant Splicing	Pathogenic	1
HNF1A	c.1136C>G	p.(Pro379Arg)	Heterozygous	Missense	Likely Pathogenic	2
HNF1A	c.1136_1137del	p.(Pro379fs)	Heterozygous	Frameshift	Pathogenic	1
HNF1A	c.1137del	p.(Val380fs)	Heterozygous	Frameshift	Pathogenic	3
HNF1A	c.1205del	p.(Asn402fs)	Heterozygous	Frameshift	Pathogenic	1
HNF1A	c.1276_1277insAGGT	p.(Phe426*)	Heterozygous	Nonsense	Pathogenic	1
HNF1A	c.1330_1331del	p.(Gln444fs)	Heterozygous	Frameshift	Pathogenic	1
HNF1A	c.1340C>T	p.(Pro447Leu)	Heterozygous	Missense	Pathogenic	2
HNF1A	c.1362dup	p.(Ser455fs)	Heterozygous	Frameshift	Pathogenic	1
HNF1A	c.1456C>T	p.(Gln486*)	Heterozygous	Nonsense	Pathogenic	1
HNF1A	c.1501G>A	p.(Ala501Thr)	Heterozygous	Missense	Likely Pathogenic	1
HNF1A	c.1556C>T	p.(Pro519Leu)	Heterozygous	Missense	Pathogenic	1
HNF1A	c.1623G>A	p.(Gln541Gln)	Heterozygous	Aberrant Splicing	Likely Pathogenic	2
HNF1A	c.1741dup	p.(Ala581fs)	Heterozygous	Frameshift	Pathogenic	1
HNF4A	c.(?_50-4517)_(*1057_?)del	p.0	Heterozygous	Whole gene deletion	Pathogenic	1
HNF4A	c.-178A>G	p.0?	Heterozygous	Regulatory	Likely Pathogenic	1
HNF4A	c.-181G>A	p.0?	Heterozygous	Regulatory	Likely Pathogenic	1
HNF4A	c.21_22del	p.(Leu8fs)	Heterozygous	Frameshift	Pathogenic	1
HNF4A	c.148T>C	p.(Tyr50His)	Heterozygous	Missense	Likely Pathogenic	1
HNF4A	c.150C>G	p.(Tyr50*)	Heterozygous	Nonsense	Pathogenic	1
HNF4A	c.199C>T	p.(Arg67Trp)	Heterozygous	Missense	Pathogenic	1
HNF4A	c.200G>A	p.(Arg67Gln)	Heterozygous	Missense	Pathogenic	2
HNF4A	c.305G>A	p.(Gly102Asp)	Heterozygous	Missense	Likely Pathogenic	1
HNF4A	c.320C>A	p.(Ala107Asp)	Heterozygous	Missense	Pathogenic	1
HNF4A	c.322G>A	p.(Val108Ile)	Heterozygous	Missense	Pathogenic	1

HNF4A	c.334C>T	p.(Arg112Trp)	Heterozygous	Missense	Likely Pathogenic	1
HNF4A	c.335G>A	p.(Arg112Gln)	Heterozygous	Missense	Pathogenic	1
HNF4A	c.340C>T	p.(Arg114Trp)	Heterozygous	Missense	Pathogenic	8
HNF4A	c.341G>A	p.(Arg114Gln)	Heterozygous	Missense	Pathogenic	3
HNF4A	c.421C>T	p.(Arg141*)	Heterozygous	Nonsense	Pathogenic	1
HNF4A	c.469A>C	p.(Lys157Gln)	Heterozygous	Missense	Likely Pathogenic	1
HNF4A	c.482del	p.(Ser161fs)	Heterozygous	Frameshift	Pathogenic	1
HNF4A	c.530T>C	p.(Val177Ala)	Heterozygous	Missense	Likely Pathogenic	1
HNF4A	c.572del	p.(Leu191fs)	Heterozygous	Frameshift	Pathogenic	1
HNF4A	c.577G>T	p.(Asp193Tyr)	Heterozygous	Missense	Pathogenic	1
HNF4A	c.691C>T	p.(Arg231Trp)	Heterozygous	Missense	Pathogenic	1
HNF4A	c.740T>C	p.(Leu247Pro)	Heterozygous	Missense	Pathogenic	2
HNF4A	c.805G>C	p.(Ala269Pro)	Heterozygous	Missense	Likely Pathogenic	1
HNF4A	c.823C>T	p.(Pro275Ser)	Heterozygous	Missense	Likely Pathogenic	1
HNF4A	c.881A>C	p.(Gln294Pro)	Heterozygous	Missense	Likely Pathogenic	1
HNF4A	c.918T>G	p.(Tyr306*)	Heterozygous	Nonsense	Pathogenic	1
HNF4A	c.925C>T	p.(Arg309Cys)	Heterozygous	Missense	Pathogenic	2
HNF4A	c.1040T>C	p.(Leu347Pro)	Heterozygous	Missense	Likely Pathogenic	1
GCK	c.45+1G>T	p.?	Heterozygous	Aberrant Splicing	Pathogenic	1
GCK	c.74T>G	p.(Leu25Arg)	Heterozygous	Missense	Likely Pathogenic	1
GCK	c.122T>C	p.(Met41Thr)	Heterozygous	Missense	Pathogenic	1
GCK	c.149A>T	p.(His50Leu)	Heterozygous	Missense	Likely Pathogenic	1
GCK	c.162T>G	p.(Ser54Arg)	Heterozygous	Missense	Likely Pathogenic	1
GCK	c.183C>A	p.(Tyr61*)	Heterozygous	Nonsense	Pathogenic	1
GCK	c.181_183delinsCAA	p.(Tyr61Gln)	Heterozygous	Missense	Likely Pathogenic	1
GCK	c.238G>A	p.(Gly80Ser)	Heterozygous	Missense	Likely Pathogenic	1
GCK	c.297G>A	p.(Trp99*)	Heterozygous	Nonsense	Pathogenic	1
GCK	c.356C>A	p.(Ala119Asp)	Heterozygous	Missense	Likely Pathogenic	1
GCK	c.389T>C	p.(Ile130Thr)	Heterozygous	Missense	Likely Pathogenic	1

GCK	c.435_436dup	p.(Leu146fs)	Heterozygous	Frameshift	Pathogenic	1
GCK	c.449T>C	p.(Phe150Ser)	Heterozygous	Missense	Pathogenic	3
GCK	c.458C>A	p.(Pro153His)	Heterozygous	Missense	Pathogenic	1
GCK	c.478G>A	p.(Asp160Asn)	Heterozygous	Missense	Pathogenic	2
GCK	c.478G>C	p.(Asp160His)	Heterozygous	Missense	Pathogenic	2
GCK	c.483+2_483+16del	p.?	Heterozygous	Aberrant Splicing	Pathogenic	2
GCK	c.540T>G	p.(Asn180Lys)	Heterozygous	Missense	Pathogenic	1
GCK	c.544G>A	p.(Val182Met)	Heterozygous	Missense	Pathogenic	1
GCK	c.556C>T	p.(Arg186*)	Heterozygous	Nonsense	Pathogenic	1
GCK	c.571C>T	p.(Arg191Trp)	Heterozygous	Missense	Pathogenic	4
GCK	c.579G>T	p.(Gly193Gly)	Heterozygous	Aberrant Splicing	Likely Pathogenic	1
GCK	c.605T>G	p.(Met202Arg)	Heterozygous	Missense	Likely Pathogenic	1
GCK	c.617C>T	p.(Thr206Met)	Heterozygous	Missense	Pathogenic	1
GCK	c.645C>G	p.(Tyr215*)	Heterozygous	Nonsense	Pathogenic	1
GCK	c.667G>A	p.(Gly223Ser)	Heterozygous	Missense	Pathogenic	1
GCK	c.676G>A	p.(Val226Met)	Heterozygous	Missense	Pathogenic	1
GCK	c.679+1G>A	p.?	Heterozygous	Aberrant Splicing	Pathogenic	1
GCK	c.704T>C	p.(Met235Thr)	Heterozygous	Missense	Pathogenic	4
GCK	c.766G>A	p.(Glu256Lys)	Heterozygous	Missense	Pathogenic	2
GCK	c.772G>A	p.(Gly258Ser)	Heterozygous	Missense	Pathogenic	1
GCK	c.772G>T	p.(Gly258Cys)	Heterozygous	Missense	Likely Pathogenic	1
GCK	c.781G>C	p.(Gly261Arg)	Heterozygous	Missense	Pathogenic	1
GCK	c.812T>C	p.(Leu271Pro)	Heterozygous	Missense	Likely Pathogenic	1
GCK	c.834C>A	p.(Asp278Glu)	Heterozygous	Missense	Likely Pathogenic	1
GCK	c.852C>A	p.(Pro284Pro)	Heterozygous	Aberrant Splicing	Likely Pathogenic	1
GCK	c.852del	p.(Gly285fs)	Heterozygous	Frameshift	Pathogenic	1
GCK	c.864-1G>A	p.?	Heterozygous	Aberrant Splicing	Pathogenic	1
GCK	c.868G>A	p.(Glu290Lys)	Heterozygous	Missense	Likely Pathogenic	1
GCK	c.878T>G	p.(Ile293Arg)	Heterozygous	Missense	Likely Pathogenic	1

GCK	c.895G>A	p.(Gly299Ser)	Heterozygous	Missense	Likely Pathogenic	1
GCK	c.895G>C	p.(Gly299Arg)	Heterozygous	Missense	Pathogenic	1
GCK	c.896G>A	p.(Gly299Asp)	Heterozygous	Missense	Pathogenic	1
GCK	c.1007C>T	p.(Ser336Leu)	Heterozygous	Missense	Likely Pathogenic	1
GCK	c.1019+2T>G	p.?	Heterozygous	Aberrant Splicing	Pathogenic	1
GCK	c.1039C>T	p.(Gln347*)	Heterozygous	Nonsense	Pathogenic	1
GCK	c.1099G>A	p.(Val367Met)	Heterozygous	Missense	Likely Pathogenic	1
GCK	c.1133C>A	p.(Ala378Asp)	Heterozygous	Missense	Likely Pathogenic	1
GCK	c.1153G>C	p.(Gly385Arg)	Heterozygous	Missense	Likely Pathogenic	1
GCK	c.1174C>T	p.(Arg392Cys)	Heterozygous	Missense	Pathogenic	1
GCK	c.1345G>A	p.(Ala449Thr)	Heterozygous	Missense	Pathogenic	1
GCK	c.1346C>A	p.(Ala449Glu)	Heterozygous	Missense	Likely Pathogenic	1
GCK	c.1358C>G	p.(Ser453Trp)	Heterozygous	Missense	Pathogenic	1
GCK	c.1364T>A	p.(Val455Glu)	Heterozygous	Missense	Pathogenic	1
ABCC8	c.617C>T	p.(Pro206Leu)	Heterozygous	Missense	Likely Pathogenic	1
ABCC8	c.2476C>T	p.(Arg826Trp)	Heterozygous	Missense	Pathogenic	2
ABCC8	c.2977C>T	p.(Arg993Cys)	Heterozygous	Missense	Likely Pathogenic	1
ABCC8	c.3547C>T	p.(Arg1183Trp)	Heterozygous	Missense	Pathogenic	1
ABCC8	c.3629G>A and 4311-2A>G	p.(Gly1210Glu) and p.?	Compound Heterozygous	Missense & Aberrant Splicing	Likely Pathogenic & Pathogenic	1
ABCC8	c.4139G>C	p.(Arg1380Pro)	Heterozygous	Missense	Likely Pathogenic	1
ABCC8	c.4139G>A	p.(Arg1380His)	Heterozygous	Missense	Pathogenic	1
ABCC8	c.4522G>A	p.(Ala1508Thr)	Heterozygous	Missense	Likely Pathogenic	1
ABCC8	c.4661G>A	p.(Arg1554Gln)	Heterozygous	Missense	Likely Pathogenic	1
ABCC8	c.4610C>T	p.(Ala1537Val)	Heterozygous	Missense	Likely Pathogenic	1
INS	c.83_88del	p.(Gln28_His29del)	Heterozygous	In-frame deletion	Likely Pathogenic	1
INS	c.137G>A	p.(Arg46Gln)	Heterozygous	Missense	Pathogenic	1
INS	c.163C>T	p.(Arg55Cys)	Heterozygous	Missense	Pathogenic	2
INS	c.254_255delinsGT	p.(Ser85Cys)	Heterozygous	Missense	Likely Pathogenic	1

INS	c.331T>G	p.(*111Gluext*?)	Heterozygous	Stop-loss	Likely Pathogenic	1
KCNJ11	c.287C>T	p.(Ala96Val)	Heterozygous	Missense	Likely Pathogenic	1
KCNJ11	c.481G>A	p.(Ala161Thr)	Heterozygous	Missense	Likely Pathogenic	1
KCNJ11	c.685G>A	p.(Glu229Lys)	Heterozygous	Missense	Pathogenic	1
KCNJ11	c.754G>A	p.(Val252Met)	Heterozygous	Missense	Likely Pathogenic	1
KCNJ11	c.964G>A	p.(Glu322Lys)	Heterozygous	Missense	Likely Pathogenic	1
NEUROD1	c.328G>A	p.(Glu110Lys)	Heterozygous	Missense	Likely Pathogenic	1
NEUROD1	c.616dupC	p.(His206fs)	Heterozygous	Frameshift	Likely Pathogenic	2
PDX1	c.217dup	p.(Leu73fs)	Heterozygous	Frameshift	Likely Pathogenic	1
PDX1	c.218del	p.(Leu73fs)	Heterozygous	Frameshift	Likely Pathogenic	1
RFX6	c.73C>T	p.(Gln25*)	Heterozygous	Nonsense	Pathogenic	2
RFX6	c.164dup	p.(Glu56fs)	Heterozygous	Frameshift	Pathogenic	1
RFX6	c.221C>G	p.(Ser74*)	Heterozygous	Nonsense	Pathogenic	1
RFX6	c.438T>G	p.(Tyr146*)	Heterozygous	Nonsense	Pathogenic	1
RFX6	c.875T>G	p.(Leu292*)	Heterozygous	Nonsense	Pathogenic	1
RFX6	c.1028T>G	p.(Leu343*)	Heterozygous	Nonsense	Pathogenic	2
MT-TL1	m.3243A>G	N/A	Heteroplasmic	N/A	Pathogenic	24
HNF1B	c.(?_1)_(*4_?)del	p.0?	Heterozygous	Whole gene deletion	Pathogenic	14
HNF1B	c.22del	p.(Leu8fs)	Heterozygous	Frameshift	Pathogenic	1
HNF1B	c.374T>C	p.(Ile125Thr)	Heterozygous	Missense	Likely Pathogenic	1
HNF1B	c.544+3_544+6del	p.?	Heterozygous	Aberrant Splicing	Pathogenic	1
HNF1B	c.(544+1_545-1)_(809+1_810-1)del	p.?	Heterozygous	Partial gene deletion (exon 3)	Pathogenic	1
GATA6	c.214G>T	p.(Gly72*)	Heterozygous	Nonsense	Pathogenic	1
INSR	c.3089G>T	p.(Gly1030Val)	Heterozygous	Missense	Pathogenic	1
INSR	c.3164C>T	p.(Ala1055Val)	Heterozygous	Missense	Pathogenic	2
INSR	c.3356G>A	p.(Arg1119Gln)	Heterozygous	Missense	Pathogenic	1
PPARG	c.1245del	p.(Phe415fs)	Heterozygous	Frameshift	Pathogenic	1
SLC29A3	c.1330G>T	p.(Glu444*)	Homozygous	Nonsense	Pathogenic	1

TRMT10A	c.79G>T	p.(Glu27*)	Homozygous	Nonsense	Pathogenic	1
WFS1	c.1885C>T	p.(Arg629Trp)	Homozygous	Missense	Pathogenic	1
WFS1	c.2053C>T and c.2254G>T	p.(Arg685Cys) and p.(Glu752*)	Compound Heterozygous	Missense and Nonsense	Pathogenic	1
WFS1	c.1107_1108insA and c.1456C>T	p.(Ala370fs) and p.(Gln486*)	Compound Heterozygous	Frameshift and Nonsense	Pathogenic	1
WFS1	c.1433G>A	p.(Trp478*)	Homozygous	Nonsense	Pathogenic	1
WFS1	c.698_707del	p.(Leu233fs)	Homozygous	Frameshift	Pathogenic	1
WFS1	c.2020G>A and c.2170C>T	p.(Gly674Arg) and p.(Pro724Ser)	Compound Heterozygous	Missense	Pathogenic	1

Supplementary Table 5: Clinical characteristics of m.3243A>G diabetes patients diagnosed in the suspected MODY cohort and diagnosed when clinically suspected with MIDD. Data is in the format median, (IQR), n for continuous variables and n (%) for categorical variables.

**Characteristic**	m.3243A>G patients identified in a suspected MODY cohort (n=24)	Patients with m.3243A>G identified when clinically suspected of having MIDD (n=54)	*P*
Age at diagnosis (y)	28 (22-34.5), 24	33.5 (26-39), 54	0.09
Duration (y)	2 (1-7.5), 24	6 (2-15), 54	0.05
Female	18 (75%)	32 (59%)	0.21
BMI (kg/m^2)	20.7 (19.5-23.4), 19	21.6 (18.9-24.4) 42	0.89
Any other extra-pancreatic feature (excluding deafness)	0 (0%)	43 (80%)	<0.001
Deafness	2 (9%)	42 (78%)	<0.001
Neurological (seizures, epilepsy, DD, autism, LD)	0 (0%)	3 (6%)	0.33
Cardiomyopathy	0 (0%)	4 (7%)	0.22
renal disease	0 (0%)	3 (6%)	0.49
myopathy/muscle weakness	0 (0%)	2 (4%)	0.48
Retinal changes	0 (0%)	6 (11%)	0.3
Lactic acidosis	0 (0%)	2 (4%)	0.48

maternal family history of deafness	1 (4%)	35 (65%)	<0.001
mother with diabetes	17 (71%)	41 (76%)	0.53
Ethnicity (non-white)	5 (21%)	5 (9%)	0.26
HbA1c (%)	7.5 (6.6-8.2), 16	7.5 (6.9-9.1), 36	0.68
HbA1c (mmol/mol)	58 (49-66)	58 (52-76)	0.68
Insulin treated	15 (63%)	42 (78%)	0.17
Insulin alone	13	34	
Insulin with Oral Hypoglycaemic Drugs	2	8	

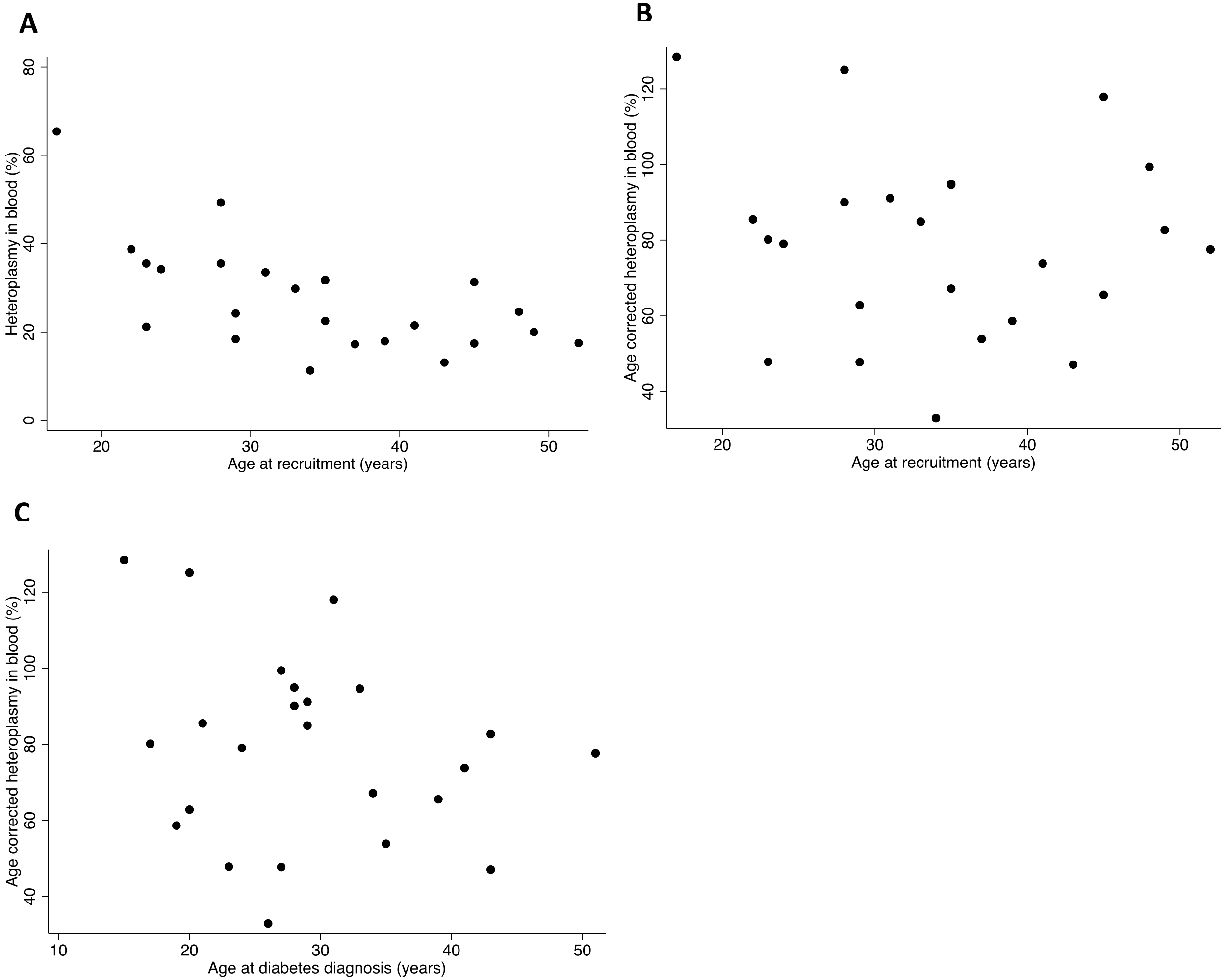

Supplementary Figure 1: Blood heteroplasmy level of m.3243A>G in patients with suspected MODY. A) Scatter graph showing percentage blood heteroplasmy against age at genetic testing. B) Scatter graph showing age-adjusted percentage blood heteroplasmy against age at genetic testing. C) Scatter graph showing age-adjusted percentage blood heteroplasmy against age at diagnosis. Blood heteroplasmy was adjusted for age using the published tool available at https://newcastle-mito-apps.shinyapps.io/m3243ag_heteroplasmy_tool/.

Supplementary Table 6: Clinical characteristics of patients with an *HNF1B* mutation diagnosed in a suspected MODY cohort and diagnosed when clinically suspected. Data is in the format median, (IQR), n for continuous variables and n (%) for categorical variables.

**Characteristic**	Patients with an *HNF1B* mutation identified in a suspected MODY cohort (n=18)	Patients with an *HNF1B* mutation identified when clinically suspected of having *HNF1B*-related disease (n=50)	*P*
Age at diagnosis (y)	17 (12-25), 18	18 (13-27), 50	0.58
Duration (y)	3.5 (1-9), 18	3 (1-8), 50	0.57
Female	11 (61%)	24 (48%)	0.25
BMI (kg/m^2)	23.2 (19.5-28.7), 16	24.4 (21.5-25.9), 28	0.97
Any extra-pancreatic feature	2 (11%)	47 (94%)	<0.001
Structural kidney disease	0 (0%)	42 (84%)	<0.001
genital tract malformations	1 (6%)	8 (16%)	0.25
Developmental delay	0 (0%)	5 (10%)	0.47
Neurological Complications (DD, Seizures, autism, LD etc)	1 (6%)	6 (12%)	0.40
exocrine pancreas deficiency	0 (0%)	8 (16%)	0.07
Gout	0 (0%)	2 (4%)	0.54
Hypomagnesemia	0 (0%)	7 (14%)	0.10
Parent with diabetes	8 (44%)	17 (34%)	0.57
Ethnicity (non-white)	3 (17%)	6 (12%)	0.69
HbA1c (%)	6.7 (6.5-11.6), 15	7.2 (6.2-8.6), 30	0.95
HbA1c (mmol/mol)	50 (48-103)	55 (44-71)	0.95
Insulin treated	14 (78%)	27 (54%)	0.10
Insulin alone	10	23	
Insulin with Oral Hypoglycaemic Drugs	4	4	
Mutation type			0.09
Missense	1 (5%)	12 (24%)	

Protein truncating (null) variant	3 (17%)	14 (28%)	
Partial or whole gene deletion	14 (78%)	24 (48%)	

Supplementary Table 7: Clinical characteristics of patients with an *HNF1B* gene deletion detected in patients with suspected MODY and patients with suspected *HNF1B*-related disease. Data is in the format median, (IQR), n for continuous variables and n (%) for categorical variables.

**Characteristic**	Patients with an *HNF1B deletion* identified in a suspected MODY cohort (n=14)	Patients with an *HNF1B* deletion identified when clinically suspected of having *HNF1B*-related disease (n=24)	*P*
Age at diagnosis (y)	17 (13-25)	20 (15-27)	0.29
Duration (y)	3.5 (1-9)	3 (1-5)	0.76
Female	9 (64%)	12 (50%)	0.50
BMI (kg/m^2)	24.6 (18.5-29.9), 12	24 (20.8-24.9), 15	0.55
Any extra-pancreatic feature	2 (14%)	23 (96%)	<0.001
Structural kidney disease	0 (0%)	18 (74%)	<0.001
genital tract malformations	1 (7%)	5 (21%)	0.38
Developmental delay	1 (7%)	4 (17%)	0.63
Neurological Complications (DD, Seizures, autism, LD etc)	1 (7%)	5 (21%)	0.38
exocrine pancreas deficiency	0 (0%)	5 (21%)	0.13
Gout	0 (0%)	0 (0%)	N/A
Hypomagnesemia	0 (0%)	4 (17%)	0.27
Parent with diabetes	3 (21%)	17 (34%)	0.65
Ethnicity (non-white)	3 (21%)	3 (13%)	0.64
HbA1c (%)	7.1 (6.5-12), 12	7.4 (6.2-8.6), 17	0.46
HbA1c (mmol/mol)	55 (48-108)	57 (44-70)	0.46

Insulin treated	11 (79%)	13 (54%)	0.17
Insulin alone	7	11	
Insulin with Oral Hypoglycaemic Drugs	4	2	

Supplementary Table 8: Clinical characteristics of patients with mutations in syndromic monogenic diabetes genes other than m.3243A>G and *HNF1B*

Gene	DNA change	Protein Change	Zygosity	Ethnicity	Age diabetes diagnosis (years)	Current Treatment	Family History	Extra-pancreatic features reported at referral	Extra-pancreatic features known to clinician but not reported at referral	Age at time of referral/age at time of follow up (years)	Additional extra-pancreatic features reported at follow-up
GATA6	NM_005257.5:c.214G>T	p.(Gly72*)	Heterozygous	White British	13	Insulin alone	Mother GDM, maternal grandfather type 2 DM.	None	None	14/17	None
INSR	NM_000208.4:c.3089G>T	p.(Gly1030Val)	Heterozygous	White British	12	None	Paternal Grandfather type 2 DM.	None	Acanthosis nigricans, PCOS.	17/22	raised C-peptide (3605pmol/L)
INSR	NM_000208.4:c.3164C>T	p.(Ala1055Val)	Heterozygous	White British	18	None	Mother, father, maternal uncle, paternal uncle and maternal grandfather type 2 DM.	None	None	33/36	Post-prandial hypoglycaemia. Normal lipid profile. Raised fasting C-peptide (576pmol/L). Mother taking U500 insulin.
INSR	NM_000208.4:c.3356G>A	p.(Arg1119Gln)	Heterozygous	White British	17	Insulin alone	Father, paternal aunt and uncle type 2 DM. Father heterozygous for the mutation.	None	None	17/19	None
INSR	NM_000208.4:c.3164C>T	p.(Ala1055Val)	Heterozygous	White British	12	OHA	father, two paternal uncles and paternal aunt type 2 DM. Father heterozygous for the mutation.	None	Acanthosis nigricans.	21/22	Normal lipid profile. Raised serum testosterone and mildly raised fasting insulin. Father has normal lipid profile and raised fasting insulin.
PPARG	NM_015869.4:c.1245del	p.(Phe415fs)	Heterozygous	Black African	19	Insulin alone	Daughter type 2 DM at age 18 years. Sister and maternal grandmother type 2 DM.	None	Partial Lipodystrophy<br>Mild acanthosis<br>Mild hirsutism<br>Irregular periods since early 30's	45/49	None
SLC29A3	NM_018344.5:c.1330G>T	p.(Glu444*)	Homozygous	South Asian	9	Insulin alone	Brother and sister with joint contractures. Sister also type 1 DM. Brother also homozygous for mutation.	Joint contractures	Not known	17/NA	Lost to follow up

TRMT10A	NM_152292.5:c.79G>T	p.(Glu27*)	Homozygous	White British	30	Insulin alone	Seven siblings with diabetes	None	Microcephaly and a severe learning disability.	57/60	None
WFS1	NM_006005.3:c.1885C>T	p.(Arg629Trp)	Homozygous	Arabic	5	Insulin alone	Father, paternal grandfather and maternal grandparents with type 2 DM	None	None	10/16	Optic atrophy and moderate sensorineural hearing loss diagnosed aged 13 years.
WFS1	NM_006005.3:c.2053C>T and NM_006005.3:c.2254G>T	p.(Arg685Cys) and p.(Glu752*)	Compound Heterozygous	White British	22	Insulin alone	Three siblings with diabetes	None	Neuropsychiatric disorder & cognitive decline. bladder instability. Ataxia & gait disturbance. Muscle weakness and neuropathy.	48/53	Hearing loss.
WFS1	NM_006005.3:c.1107_1108insA and NM_006005.3:c.1456C>T	p.(Ala370fs) and p.(Gln486*)	Compound Heterozygous	South Asian	6	Insulin alone	Maternal uncle and grandfather type 2 DM	None	Blurred vision, Optic atrophy diagnosed aged 8 years. Mild behavioural problems.	13/16	None
WFS1	NM_006005.3:c.1433G>A	p.(Trp478*)	Homozygous	South Asian	2	Insulin alone	Brother type 1 DM at age 2 years and homozygous for mutation	None	Not known	2/NA	Lost to follow up
WFS1	NM_006005.3:c.698_707del	p.(Leu233fs)	Homozygous	Arabic	8	Insulin alone	Sister type 1 DM and deafness at age 7 years	None	None	12/13	Bilateral optic atrophy and diabetes insipidus.
WFS1	NM_006005.3:c.2020G>A and NM_006005.3:c.2170C>T	p.(Gly674Arg) and p.(Pro724Ser)	Compound Heterozygous	East Asian	17	OHA + Insulin	Maternal & paternal grandparents' type 2 DM	None	Bilateral optic atrophy.	19/20	Mild dyskinesia and abnormal reflexes.
